# Reply to comments by Olson *et al*. 2017 and Stien 2017

**DOI:** 10.1098/rspb.2017.1743

**Published:** 2017-11-22

**Authors:** Guillaume Chapron, Adrian Treves

**Affiliations:** 1 Grimsö Wildlife Research Station, Department of Ecology, Swedish University of Agricultural Sciences, 73091 Riddarhyttan, Sweden; 2 Nelson Institute for Environmental Studies, University of Wisconsin, 30A Science Hall, 550 North Park Street, Madison, WI 53706, USA

The management of large carnivores remains a contentious issue in many countries. Among the most contentious management options is ‘tolerance hunting’, or the killing of predators to increase tolerance among groups of people who do not accept the presence of these animals [1,2]. In [3,4], we used Bayesian state space models to evaluate the hypothesis that liberalizing culling of wolves changed wolf population dynamics from 1995 to 2012, and concluded it slowed growth, which we inferred was owing to increased poaching. Olson *et al*. [5] and Stien [6] re-visit our paper and we address their criticisms below.

First, we disagree with Olson *et al*.'s [5] and Stien's [6] assertions that our paper ignores the literature or reports it in a biased manner. We simply disagree about the interpretation of the literature as we explain below. While they can have a different interpretation of those papers, it does not mean that ours is incorrect and Stien's [6, p. 1] phrasing ‘biased reporting of previously published results’ almost suggests intent from us to mislead the reader. Both Olson *et al*. [5] and Stien [6] raised the issue of density dependence analysed by Stenglein *et al*. [7]. In that paper, the information on density dependence relevant to our paper is in figures 3, S2.4, S2.5 and S2.6 (we cannot find reported numerical estimates on how recruitment changed during the relevant period for our study in [7]). Stenglein *et al*. [7, p. 5] wrote that ‘The evidence for a negative slope of the line for *t* > 18 was 69.0% (proportion of posterior that was <0)’ but this concerns all years post-1998, which also include many years without culling. For the relevant period for our paper (when culling was allowed or wolf years 2004–2012), we need to interpret the figures ourselves. On figures 3, S2.4, S2.5 and S2.6 in [7], we find no obvious difference between the confidence intervals of annual recruitment estimates. In fact, the only significant drop in recruitment seems to happen much earlier, at the beginning of the *t* > 18 period (1998–2001 approximately) whereas the years with culling seem to show a stable recruitment regardless of the models used [7]. Because Stenglein *et al*. [7] clearly concluded that they found no density dependence on survival, we observed then and still interpret Stenglein *et al*. [7] to show no density dependence for the period relevant to our study. An additional sentence in our discussion in [3] explaining what we just explained above might have been welcome but seemed a digression. We also chose not to mention that Stenglein *et al*. [7, p. 5] appear to trust their model because ‘48.4% of the time, the estimated population sizes in Wisconsin from 1981 to 2011 were within the 95% posterior intervals of *μ_t_*’ implying that more than half the time their estimates failed this relatively undemanding test. Stenglein *et al*. [7] also did not, in our opinion, properly handle uncertainty by using the midpoint between minimum and maximum population size as their population count (while we allowed fluctuations between minimum and maximum in [3]). Both Olson *et al*. [5] and Stien [6] further insist that the decline in growth rate is owing to negative density dependence. Olson *et al*. [5] present a compilation of studies, but which also includes some unrelated to negative density dependence (see our electronic supplementary material). Neither of those papers present, in our opinion, empirical evidence to support a mechanism for density dependence in the population and period under discussion. Stien [6] argues that the quadratic relationship he found for area against population size is evidence of negative density dependence. However, as we wrote previously [8], one must first demonstrate a mechanism to assert negative density dependence. Indeed, the United States Fish and Wildlife Service reported that the Wisconsin wolf population grew from minima of 746 to 866 by April 2016 [9] after all wolf-killing including tolerance hunting was barred in December 2014, or a 1-year growth of 16%, which is larger than the annual median growth during our study period. This accelerating growth at the relevant population size demonstrates that there is still no evidence consistent with negative density dependence in the Wisconsin wolf population during the period of interest for our study.

Olson *et al*. [5] also argue that their previous study [10] demonstrated that illegal killing decreases with increasing availability of lethal management. However, this study [10] was, in our opinion and that of an anonymous reviewer, not quantitatively rigorous. One reviewer of our paper [3] indeed agreed and wrote that our ‘paper is also important because the results are at least somewhat contradictory to a recent paper Olson *et al.* [10]. That recent paper had some important shortcoming for which this paper seems to “fix”’. We admit we might have explained the below shortcomings in our original paper [3] but did not wish to appear confrontational. Olson *et al*. [10] assumed that observed poaching correlated tightly to unobserved poaching (even for radio-collared wolves). Embracing this assumption leads to the faulty conclusion that observed poaching is an unbiased sample of all poaching and can be used as the response variable for a correlation with temporal changes in policy. Treves *et al*. [11] did not find support for that assumption. In a separate study in Scandinavia, Liberg *et al*. [12] found that two thirds of poaching was not observed. For Wisconsin wolves, Treves *et al*. [11] estimated that same observation error to be half of all poached wolves. Olson *et al*. [10] also used the number of recovered radio-collared wolves inferred to have died from poaching as their response variable, without considering errors in inferring poaching as a cause of death. Systematic errors in attributing poaching to Wisconsin wolf carcasses ranged from 6–37% depending on which subsample one examined, as reported by veterinary pathologists contributing to Treves *et al*. [13]. Both Olson *et al*. [10] and Treves *et al*. [11,13] agree that a high proportion of radio-collared wolves disappeared without trace (unknown fate), which must be addressed in some way in any analysis of poaching [11]. Most importantly, Olson *et al*. [10] ignored exposure time of radio-collared wolves. We do not understand why they did not use a survival (time to event) model with the proportion of the year with culling as an explanatory variable. However, even using a time to event model would require a proper treatment of unknown fates. Finally, Olson *et al*. [10] did not seem to consider that marked animals (radio-collared wolves) may not suffer the same mortality pattern as the unmarked population. This has been shown specifically in two recent studies of wolves, which have undermined the assumption of identical mortality patterns [14,15].

Olson *et al*. [5] and Stien [6] raise other points which we address in detail in our electronic supplementary material. Briefly, Stien [6] claims that there is a strong link between probability of reproduction and proportion of the year with legal culling. However, we believe other models in Stien [6] supplementary code do not support this conclusion, which, if they would, would still not warrant a change of our conclusions (see electronic supplementary material). We explain Olson *et al*. [5]'s assertion—that our hypothesis is not parsimonious—is built on a misunderstanding of the cause-and-effect relationships between cognition and behaviour. Moreover, Olson *et al*. [5]'s hypothesis of density dependence is not supported by evidence (see above), so its simplicity does not give it strength. We also argue that there is no support for the frustration hypothesis proposed by Olson *et al*. [5] because previous research demonstrates that tolerance for wolves declined, and inclination to poach rose, in the years following culling authority. Here and elsewhere, the reasoning in Olson *et al*. [5] leaves the impression of cherry-picking the literature while accusing us inaccurately of ignoring or misrepresenting it. Olson *et al*. [5] insinuate that we chose to start our analysis in 1995 because it somehow supported our hypothesis. Our choice is justified by two of Olson *et al*.'s [5] co-authors writing how monitoring substantially improved after 1995 [16]. The papers they cite [7,17,18] that begin analyses earlier do not seem to account for that change in census methods, which may affect their results. Finally, Olson *et al*. [5] criticize us for calling our study ‘quasi experimental’ and write that it is instead a ‘worst case design’ despite having published on the exact same study system [10]. We do not follow the logic by which a system can suddenly become the worst when other different authors write about it. Overall, the pattern emerging from analyses in Olson *et al*. [5,10] is one of a stream of unrigorous assertions which together portray a picture of the Wisconsin wolf population that is inaccurate. When management policies are built on such weak assertions, these policies cannot have a scientific basis, as has been shown for wolf hunting in the United States [19]. In addition, Olson *et al*. [5] seem, in our opinion, inclined to divert from a collegial discussion and adopt the language and style of advocacy. While there may be many reasons to pledge allegiance to management agencies, we believe that scholarly debates are not compatible with ad-hominem attacks and misleading soundbites.

We appreciate the scrutiny that our analysis and our writing have sparked. Science progresses through invalidation of hypotheses and presentation of new evidence, therefore we welcome scrutiny of our work and collegial discussions. However, we also feel obligated to point out that statements supporting the tolerance hunting hypothesis, either from scientists or governments, seem to be taken for granted and evade scrutiny. A recent illustration is a paper about wolves in Norway bluntly claiming that ‘it is not an unreasonable expectation that allowing legal harvest might prevent some of the illegal killing’ [20, p. 135]. In our opinion, the careful wording of nuances in the above sentence only signals a value-based statement intended to influence policy regardless of evidence. While our model has faced substantial and legitimate scrutiny, scientists have remained silent about flaws or lack of evidence supporting the tolerance hunting hypothesis. In other words, killing predators appears immune to evidence-based scrutiny, while not killing predators must be justified by the highest level of evidence. One possible reason is that killing predators may simply be viewed as not worthy of justification unless one is driven by emotions [21], an attitude revealing contempt for changing public attitudes about the value of wildlife [22] and a refusal to serve the broad public interest [23]. Another possible reason may be that killing predators is a goal by itself regardless of its effectiveness in reducing poaching because it provides political services [24]. As a consequence, tolerance hunting is today a widespread management intervention for large carnivores [2] (see our electronic supplementary material for updated context), perhaps because it has the potential to justify large scale killing and is extremely difficult to evaluate scientifically. We believe that double standards in evaluating evidence are hazardous. The double standard that we observe runs contrary to the precautionary principle and the level of scrutiny should not be lower or plainly absent for writings supporting tolerance hunting than for results invalidating it. We conclude by hoping that the debate our paper triggered will encourage further research on this controversial topic.

## Supplementary Material

Supplementary Material

## Data accessibility

This article has no additional data.
