# Supplementary material for: Reply to comments by Olson et al. 2017 and Stien 2017
Source: Proc Biol Sci. 2017 Nov 22;284(1867):20171743. doi: 10.1098/rspb.2017.1743 (PMC5719172; doi:10.1098/rspb.2017.1743)
Supplement: Supplementary Material [file rspb20171743supp1.docx]

**Supplementary Material**

Density-dependence in the Wisconsin wolf population

Olson et al. [5] compile studies that they report as having documented negative density dependence, but we find that their list includes a study that documented positive density dependence (Allee effect) in Wisconsin [17] and another study of Michigan and Wisconsin describing an individual based model and not including the term “density dependence” [25]. Moreover, the studies they list are North American focused and ignore relevant studies from other regions of the world. For example, wolves in Western Poland have grown exponentially with no sign of density dependence [26]. In addition, their list includes populations such as the tiny Isle Royale National Park and the vast protected areas of Yellowstone National Park, neither of which seems a reasonable comparison to the Wisconsin and Michigan wolf populations in their heavily human dominated landscapes without large protected areas. All of these observations raise concerns about the criteria Olson et al. [5] used to build their list. As we wrote previously [8], fitting a logistic growth model alone cannot demonstrate density dependence as was done in [18]. Specifically, a mechanism must be found to support a claim of density dependence. This applies also to Stien [6]’s writing that the quadratic relationship he found for area against population size is evidence of negative density- dependence. It is not (see section: Relationship for area against population size in this supplement). Many of the studies Olson et al. [5] report (in their Table S1) do not present evidence for a mechanism. Still, when Olson et al. [5] report mechanisms for density dependence, these are not found for the Wisconsin wolf population: they propose litter size would decrease [27], but Stenglein et al. [7] shows no negative density-dependence on recruitment during the period of our study (see main text), they propose reproduction would decrease [27], we find it increases, they propose survival would decrease [28], Stenglein et al. [7] shows it remained constant. Finally, we present evidence in the main text about continued population growth above the median annual rate at the same time as the wolf population apparently exceeded the densities we studied.

Chain of inference and parsimonious hypotheses

Olson et al. [5] argue that our hypothesis is not parsimonious because it requires a longer chain of inference. They do so by presenting their own interpretation of three different hypotheses and their respective chains of inference. Our hypothesis is presented with the longest chain, while density dependence has the shortest one (one link only). We find this misleading. For one, they misunderstand the cause-and-effect relationships between cognition and behavior [29]. For both the frustration hypothesis and our hypothesis – that the policy signal of legalizing culling motivated poachers – there can be a single cognitive step and a single behavioral step. For the former, the cognitive step is that would-be poachers are motivated by frustration with continued protection of wolves, whereas our hypothesis proposed would-be poachers are motivated by lessening protections. The causal chains are the same length. In addition, Olson et al. [5] should also increase the chain for density dependence by presenting at least one falsifiable, detailed biological mechanism, because density dependence is not as simple as “*density dependence in component of growth → reduced growth at high density*”. Finally, a hypothesis that is not supported by evidence does not gain strength by being simpler.

Frustration hypothesis

Olson et al. [5] assert that “*there is no evidence that devaluation of wolves was exclusive to the period after implementation of LDM [2003]*”. First, we never said this was exclusive, rather we substantiated a decrease in tolerance for wolves, during the period 2001–2013. Second, AT and his colleagues have meticulously measured changes in human attitudes in the same individual residents of Wisconsin’s wolf range since 2001. Their measures in four separate surveys include the years 2001, 2004, 2009, and 2013 as well as a focus group approach with repeated measures from 2010-2012. Four publications [30-33] demonstrate that tolerance for wolves declined following culling authority and three measured inclination to poach, which also rose in those years. Indeed, Hogberg et al. [31] document that frustration continued to increase after wolf-killing was further liberalized to include public hunting and trapping. The non peer-reviewed report [34] on attitudes that Olson et al. [5] cite cannot address the topic of change in attitudes because it is a measure at one time point. Likewise, the Michigan state agency report [35] cited by Olson et al. [5] was not a measure of change in attitudes either. We still find no evidence in support of the frustration hypothesis in [5] and emphasize that the highest tolerance for wolves was measured in 2001 [32] and ever since it has declined in Wisconsin, when we compared the same individuals’ responses to the same questions years later [33]. The focus group study [30] seems to directly invalidate the frustration hypothesis.

Relationship for area against population size

Stien [6] argues that our log linear model is not appropriate to detect density dependence and that a quadratic relationship for area against population size is needed. Stien [6] writes that the quadratic relationship he found is evidence of negative density-dependence. We are not convinced and we reassert that one must also show that a life history parameter affecting birth or death exists before claiming density-dependent population growth. The lack of empirical evidence for a decline in demographic parameters with increasing population size is the crux of the matter here. If as Stien [6] argues, weak evidence of density dependence in total area used by the entire wolf population implies density-dependence in wolf population growth, then Stien [6] must also show that the decrease resulted from density effects on life history itself rather than other factors independent of density. For example, the area used by the entire wolf population does not necessarily bear a direct, causal relationship to pressures acting on individual wolf packs. In addition, the data on area used by the entire wolf population does not have an observation error which means that it is unclear whether the small, reported 0.0016 wolf / km^2^ increase from 2000–2011 is evidence of increasing density. Stien [6] has not presented a single such density-related biological change, nor explained how density changed if at all. We are not persuaded by the argument in Stien [6] for density-dependence based on the total area used by the wolf population because without associated changes in survival or reproduction, it is unclear then how this assumed density-dependence can account for a decline in growth rate that we reported. There may be changes in behavior but if those changes do not translate into changes in demographic parameters, it is difficult to understand how they can change growth rate. Finally, we point out that Figure 1 in Stien [6] is strongly influenced by the last data point when the area grew less but the population still grew 13% which is the median growth in the time series.

Probability of reproduction as a function of proportion of the year with legal culling

Stien [6] models the probability of reproduction as a function of proportion of the year with legal culling (see associated Figure 2 in Stien [6] ). Specifically, Stien [6] mentions one model “*slope = -0.89, SE = 0.41, P = 0.03, binomial GLMM with logit link function and year fitted as random effect*”. However, there are also other models that have very close AIC values where the proportion of the year with legal culling is not significant or is absent (see models lme.breed.H and lme.breed.DH in Stien [6] supplementary material) but that Stien [6] did not discuss. Stien [6] writes in his conclusion that “*My analysis highlights the responsibility that researchers have to expose models to alternative hypotheses that are refined and biologically plausible*”. We agree and add that models explored in Stien’s R code [6] merit further consideration. When these models are considered, it is much less certain that the negative correlation between reproductive rates and legal culling is so important. In our opinion, after assessing the model including both variables and the model-averaged coefficients, neither the proportion of the year with legal culling nor the number of wolves culled are significant predictors of probability of reproduction. Both independent variables together show low multicollinearity after testing a variance inflation factor (VIF = 1.76), so it is statistically correct to include both variables in the model to explain the change in probability of reproduction. Indeed, we may lose valuable statistical and biological information by focusing only on proportion of the year with legal culling while ignoring the number of wolves culled or ignoring the alternative models. In addition, even if Stien’s [6] claim of a strong link between probability of reproduction and proportion of the year with legal culling were right, this would not change our message [3, 4]*.* Reproductions might decrease if culling and poaching killed pack members that bred or supported reproduction. So in our opinion, Figure 2 and Stien’s [6] assertion that “*reproductive rates have to be accounted for in the analysis of population growth rates*” do not invalidate our conclusion and might on the contrary suggest a mechanism through which the policy signal operates. Our original paper [3] ended with an implicit hypothesis from our inference that poaching rose; it is possible that poaching of breeding adults in particular rose or that poaching of pups pre-census rose. Similarly, Stien [6] concluded from his extension of our Bayesian model that “*there was a strong positive relationship between probability of reproduction in year t and the population growth rate from year t-1 to t*” [6], which is not relevant for our analysis. It only shows that a population grows more when it breeds more, but does not evaluate the role of the policy signal (the proportion of the year with legal culling) in changing growth over time.

Updated context on tolerance hunting

To provide more updated context, the issue of hunting large carnivores to increase people’s tolerance has become white hot in many countries. In the USA, the FWS final rule to delist the grizzly bear [36] states that “*removals promote conservation of the GYE grizzly bear population by minimizing illegal killing of bears*” without providing evidence and adds that “*based on recent experiences with wolves in Idaho and Montana, social tolerance for wolves improved as both States implemented an adaptive management approach to managing conflict during the post-delisting monitoring period”*, apparently confusing tolerance for wolves and tolerance for authorities managing wolves and mischaracterizing the research as revealing changes in tolerance – if they are citing the only such study of which we are aware [37]. In Norway, the government faced a political controversy in winter 2016–2017 after the environment minister decided there was no valid reason to kill 47 wolves out of a population of 68 wolves and scaled down the hunting quota to 15 [38, 39]. In Finland, after two years of such tolerance hunting intended to reduce poaching, the wolf population has collapsed (by 25% in 2017, down to 150–180 animals) [40, 41] while a recent analysis of the dynamic of wolf poaching in Finland concluded that “*tolerance for carnivores cannot be promoted by legal hunting alone*” [42]. These controversies are likely to continue if governments and scientists continue to advocate for tolerance hunting without evidence.

Supplementary references

25. Stenglein, J.L., et al., *An individual-based model for southern Lake Superior wolves: A tool to explore the effect of human-caused mortality on a landscape of risk.* Ecological Modelling, 2015. **302**: p. 13-24.

26. Nowak, S. and R.W. Mysłajek, *Wolf recovery and population dynamics in Western Poland, 2001–2012.* Mammal Research, 2016: p. 1-16.

27. Stahler, D.R., et al., *The adaptive value of morphological, behavioural and life‐history traits in reproductive female wolves.* Journal of Animal Ecology, 2013. **82**(1): p. 222-234.

28. Cubaynes, S., et al., *Density‐dependent intraspecific aggression regulates survival in northern Yellowstone wolves (Canis lupus).* Journal of Animal Ecology, 2014. **83**(6): p. 1344-1356.

29. Ajzen, I., *The theory of planned behavior.* Organizational behavior and human decision processes, 1991. **50**(2): p. 179-211.

30. Browne-Nuñez, C., et al., *Evaluating the potential for legalized lethal control of wolves to reduce illegal take: A mixed-methods examination of attitudes and behavioral inclinations.* Biological Conservation, 2015. http://dx.doi.org/10.1016/j.biocon.2014.12.016.

31. Hogberg, J., et al., *Changes in attitudes toward wolves before and after an inaugural public hunting and trapping season: early evidence from Wisconsin's wolf range.* Environmental Conservation, 2015. **FirstView**: p. 1-11.

32. Naughton-Treves, L., R. Grossberg, and A. Treves, *Paying for tolerance: the impact of livestock depredation and compensation payments on rural citizens’ attitudes toward wolves.* Conservation Biology, 2003. **17**(6): p. 1500-11.

33. Treves, A., L. Naughton-Treves, and V. Shelley, *Longitudinal Analysis of Attitudes Toward Wolves.* Conservation Biology, 2013. **27**(2): p. 315-323.

34. Holsman, R., N. Kaner, and J. Petchenik, *Public attitudes towards wolves and wolf management in Wisconsin*. 2014, Wisconsin Department of Natural Resources Madison, WI.

35. Beyer, D., et al., *Review of social and biological science relevant to wolf management in Michigan.* Michigan Department of Natural Resources, Lansing, Michigan, USA, 2006.

36. USFWS, *Removing the Greater Yellowstone Ecosystem Population of Grizzly Bears From the Federal List of Endangered and Threatened Wildlife.* Federal Register, 2017. **82 FR 30502**.

37. Lewis, M.S., et al., *Selected results from four separate surveys of resident Montanans regarding Montana’s wolf hunt.* HD Unit Research Summary, 2012(33).

38. Harvey, F., *Norway plans to cull more than two-thirds of its wolf population.* The Guardian, 2016. https://www.theguardian.com/environment/2016/sep/16/norway-wolf-cull-government-wwf-friends-earth-environment-protest.

39. Sutterud, T. and E. Ulven, *Norway reprieves 32 of 47 wolves earmarked for cull.* The Guardian, 2016. https://www.theguardian.com/environment/2016/dec/20/norway-reprieves-32-of-47-wolves-earmarked-for-cull.

40. AFP, *Finland approves wolf hunt in trial cull.* The Guardian, 2016. https://www.theguardian.com/world/2016/jan/21/finland-approves-wolf-hunt-trial-cull-illegal-poaching.

41. Harvey, F., *Finland has far fewer wild wolves than previously thought, census shows.* The Guardian, 2017. https://www.theguardian.com/world/2017/jun/26/finland-has-far-fewer-wild-wolves-than-previously-thought-census-shows.

42. Suutarinen, J. and I. Kojola, *Poaching regulates the legally hunted wolf population in Finland.* Biological Conservation, 2017. **215**: p. 11-18.
